# The presence of tomato leaf curl Kerala virus AC3 protein enhances viral DNA replication and modulates virus induced gene-silencing mechanism in tomato plants

**DOI:** 10.1186/1743-422X-8-178

**Published:** 2011-04-18

**Authors:** Kalyan K Pasumarthy, Sunil K Mukherjee, Nirupam R Choudhury

**Affiliations:** 1 International Centre for Genetic Engineering and Biotechnology, Aruna Asaf Ali Marg, New Delhi-110067, India

## Abstract

**Background:**

Geminiviruses encode few viral proteins. Most of the geminiviral proteins are multifunctional and influence various host cellular processes for the successful viral infection. Though few viral proteins like AC1 and AC2 are well characterized for their multiple functions, role of AC3 in the successful viral infection has not been investigated in detail.

**Results:**

We performed phage display analysis with the purified recombinant AC3 protein with Maltose Binding Protein as fusion tag (MBP-AC3). Putative AC3 interacting peptides identified through phage display were observed to be homologous to peptides of proteins from various metabolisms. We grouped these putative AC3 interacting peptides according to the known metabolic function of the homologous peptide containing proteins. In order to check if AC3 influences any of these particular metabolic pathways, we designed vectors for assaying DNA replication and virus induced gene-silencing of host gene *PCNA*. Investigation with these vectors indicated that AC3 enhances viral replication in the host plant tomato. In the *PCNA *gene-silencing experiment, we observed that the presence of functional AC3 ORF strongly manifested the stunted phenotype associated with the virus induced gene-silencing of *PCNA *in tomato plants.

**Conclusions:**

Through the phage display analysis proteins from various metabolic pathways were identified as putative AC3 interacting proteins. By utilizing the vectors developed, we could analyze the role of AC3 in viral DNA replication and host gene-silencing. Our studies indicate that AC3 is also a multifunctional protein.

## Background

Geminiviruses are circular ssDNA containing plant viruses with a genome size of ~ 2.7 kb [1]. Geminiviruses have an atypical genomic content. They are either monopartite with a single genomic component [2], monopartite with a satellite DNA that is around half the size of the genome [3] or bipartite with two genomic components of ~2.7 kb encoding different genes on both components [4]. Monopartite viruses encode all the genes required for successful infection, replication and movement on the single genome. In case of monopartite viruses with satellite DNA and bipartite viruses, the DNA A contains the genes necessary for replication while the cognate genome component encodes genes for infectivity and movement within the plants [3,5].

Whiteflies and leaf-hoppers are the vectors that transmit geminiviruses from one plant to other. These viruses replicate their DNA via rolling circle replication mechanism by utilizing the host plant cellular machinery [5-7]. Geminiviral proteins expressed after a successful viral infection in a plant cell induce the expression of host cell replication machinery from the differentiated plant cells [8-11]. The induced replication machinery is then diverted on to the viral DNA through the protein-protein interactions by the viral proteins for the productive replication [12-17].

Geminiviral proteins are often multi-functional in nature. Complementary strand of the geminiviruses encode four ORFs, viz., AC1, AC2, AC3 and AC4. Replication initiator protein (Rep/AC1/C1) is an essential viral protein for replication [18]. It binds the viral DNA in a sequence specific manner by recognizing the iterons at the origin of replication on the viral DNA [19-22]. Rep functions as a site-specific endonuclease by recognizing the hairpin loop structure and sequence at the viral origin of replication to initiate the viral replication. It also functions as a ligase to terminate the replication of viral DNA [23-27]. Rep has the unique ability to act as a repressor of its own transcription [28,29] and thereby regulates the expression of down-stream AC2 and AC3 genes [30]. Rep is also an ATPase [26,31,32] and a helicase [32,33]. In addition, it interacts with various host proteins [9,13-16,34,35] and viral proteins [36,37]. Similarly, the C2/AC2 protein of geminiviruses can bind to the DNA [38] and control the coat protein gene expression [39,40] either by activation or derepression [41]. AC2 is also known for its ability to suppress post-transcriptional gene-silencing mechanism [42-44] inside the host plant by inhibiting adenosine kinase [45,46] or by reducing genome wide cytosine methylation [47]. AC2 also inhibits SNF1 kinase to reduce the basal defense [48]. Likewise, AC4/C4 protein from geminiviruses was also shown to have multiple functions with roles in post-transcriptional gene-silencing [49,50], movement of virus inside the host cells [51,52], cell division [53], transcription [54] and interacts with host protein AtSKeta - a protein from brassinosteroid signaling pathway [55].

Such a battery of multiple functions in viral proteins is most of the time brought out by their ability to form hetero-oligomer or homo-oligomer. In case of the geminiviral proteins, Rep/AC1 is able to bind, nick and ligate DNA as a monomer. However, its helicase activity is strictly dependent on its ability to form a higher order homo-oligomer [32,33]. One possible way by which Rep is able to induce the replication machinery is through formation of a hetero-oligomer by interacting with retinoblastoma protein [9,56]. Similarly, AC2 protein is capable of interacting with ADK and suppresses local gene-silencing as a monomer whereas it can transactivate the virion sense strand genes as an oligomer only [57]. These observations indicate that the ability to form oligomers and to interact with other host proteins confers unique properties to the viral proteins which they cannot perform as monomers.

AC3 protein was shown to interact with viral protein AC1 [36,58]. It was also shown to interact with host proteins like pRBR [12], PCNA [14] and SlNAC1 [59]. AC3 was shown to enhance viral DNA replication by an unknown mechanism [60-65]. Preliminary studies on AC3 oligomerization suggested that AC3 also forms a higher order oligomer like AC1 [58,66]. Together, these hetero and homo-oligomerization studies observed in case of AC3 suggest that it might also have multiple functions in addition to its role in replication which is unexplored as yet. In this study we tried to address the roles of Tomato leaf curl Kerala virus-[India:Kerala II:2005] (DQ85263) AC3 protein in the viral life cycle. We have performed an exhaustive phage display analysis to find out the interacting peptides of AC3 protein. These interacting peptides were observed to be homologous to proteins from various metabolisms indicating the likely role of AC3 in these cellular pathways. Since replication of viral DNA and gene-silencing are the two important phenomena that determine the progress of viral infection, we have chosen to investigate the role of AC3 in these biological processes. We have designed vectors to analyze the role of AC3 in replication and virus induced gene-silencing in both yeast and plants.

## Results and Discussion

### Phage display analysis for AC3 interacting peptides

AC3 protein of geminiviruses is a highly hydrophobic protein containing around 62% aromatic amino acids [58]. This property poses difficulty in isolating the AC3 protein (with small tags or without tag) in the soluble fraction in sufficient quantities from bacterial cells [67]. Although it is possible to express the TGMV-AC3 protein in soluble fraction in insect cell lines but purification in high quantities becomes uneconomical [36]. Bioinformatic analysis indicated that AC3 proteins lack similarity to any known enzymatic motifs [58,68]. All these factors hindered the exploration of the mechanistic role of AC3 on enhancing viral DNA replication and the existence of any other role in viral infection. In order to find the AC3 interacting peptides which could indirectly point towards the likely role of AC3 in other cellular processes, we have employed phage display analysis.

Since AC3 protein could not be isolated in the soluble fraction without the MBP fusion tag, we have performed the phage display analysis with MBP-AC3, using MBP as a control. The unique peptides that were observed with the MBP-AC3 but not with MBP were considered for further analysis (Tables 1,2,3, &4, Additional file 1a). Homology search of these peptides against *Arabidopsis thaliana *protein database was performed to identify the putative AC3 interacting proteins. We noticed two proteins, namely pRBR and GRIK1/GRIK2 proteins (Table 3), which are well known to interact with geminiviral protein Rep [35,69]. pRBR interacts with AC3 also [35]. The peptide regions interacting with both these proteins are four residues in length. Thus we included the list of proteins with homology of at least four residues in length in shortlisting the putative interacting proteins along with their E-values. We have taken the E-value of pRBR as the threshold value for short listing various proteins. Further, we have included only those proteins with at least two or more hits from the same peptide or from different peptides identified in the phage display. Those proteins with an E-value less than that of GRIK1 were also included even if they have only one hit from the phage peptide (Tables 1,2,3, &4).

**Table 1:** Putative interacting proteins of ToLCKeV AC3 from RNAi pathway

Peptide	Protein	Accession Number	Start	Interacting Sequence in Peptide(s)	E-value
AVGGQTPIRAKI	Repressor of Silencing 1 (ROS1)	Q9SJQ6.2	79	**GQTPI**	517

NAISWFPMHLAH	Suppressor of gene silencing 3 (SGS3)	NP_197747.1	237	**AI**S**WF**P**MH**PL**LAH**	214

YALKHLPESTIP	Hua Enhancer 1 (HEN1)	AAL05056.1	704	**YALKHI**R**ES**	20

AYSPISTVTQPY	(HEN4)	AAO37828.1	403	**AY**GR**PI**E**TMTQ**	517
		AAO37829.1	858	**TVTRPY**	517

APGYARLPSLMS	Dicer-like 1 (DCL1)	NP_171612.1	687	**LPSL**	7294
		Q9SP32.2	948	**PGTAR**	42568
		NP_171612.1	1329	**RLPSIM**	287

SMTHLYTDLWQP WHKHIPSPRASS	Dicer-like 2 (DCL2)	NP_001078101.1 NP_566199.4	29	**HQYTDL**	694
			246	**IPSP**K**RAS**	214
			1224	**HKHI**	1677
			1235	**HKHI**	1677

NVHIRQPLGASS	Argonaute1 (AGO1), AGO6	AAB91987.1	47	**NV**S**VRQP**	932

NISSIRPTLVEV	AGO1	NP_175274.1	129	**VSS-QPTL**S**EV**	1603
			366	**SIRPT**	517
			649	**S**A**RP**EQ**VE**	54615

SMTHLYTDLWQP	AGO2	NP_174413.2	910	**TH**Y**YT-LW**	694

WHKHIPSPRASS LLHAPYDHSVSP	AGO7, Pinhead like protein, zippy	NP_177103.1 AAG60096.1 AC073178_7	706	**SMT**H**LY**	694
			148	**W**N**K**K**IP**T**P**	386
			14	**KHIPS**	386
			25	**LLH**K**PY**H**H**H**V**	214
			75	**HN**S**LPP**P**PP**	7294
			80	**PPPPP**H**L**	1677
			91	**PPLPP**L**L**	160
			98	**PLPP**	3020
			184	**YN**VE**ISP**	137978
			293	**PLPPE**	2250

The details of phage display identified peptides, proteins with the homologous regions, accession number of the proteins, starting co-ordinate of the matching region in the protein sequence, matching sequence in the phage peptide and the E-value of the corresponding match are shown. Residues in bold are identical (or similar in few cases) to the residues in the protein sequence. Mismatches to the protein sequence are shown in reduced font size.

**Table 2:** List of putative ToLCKeV AC3 interacting DNA and histone modifying enzymes

Peptide	Protein	Accession Number	Start	Interacting Sequence in Peptide(s)	E-value
IQSGTPHPPLRS	H3-K9 Methyltransferase	NP_565056.1	87	**PPLRS**	517
			26	**PLRS**	5436

AMYYPLWPSLVY HLPRHHWQWPSR	Histone acetyl transferase	NP_173115.1	562	**QWPS**	890
			986	**AMYY**	517

LEAPRPTPAVPM	Variant in methylation 2 (VIM2), VIM3, VIM4, VIM5	NP_176091.2 NP_176779.2 NP_176778.1	448	**PRPLPNVP**	517

HILSPSGSPRMS	MOM	NP_563806.1	394	**I**P**SPSG**	9787
			1588	**PSGS**	13133
			1821	**SPSGAPR**	119

GSAVASTLPLGQ	Decreased methylation to DNA (MET1)	NP_199727.1	522	**AVASTL**	287
			1181	**STLPLPGQ**	287

The details of phage display identified peptides, proteins with the homologous regions, accession number of the proteins, starting co-ordinate of the matching region in the protein sequence, matching sequence in the phage peptide and the E-value of the corresponding match are shown. Residues in bold are identical (or similar in few cases) to the residues in the protein sequence. Mismatches to the protein sequence are shown in reduced font size.

**Table 3:** List of putative ToLCKeV AC3 interacting proteins from DNA recombination and cell cycle pathway

Peptide	Protein	Accession Number	Start	Interacting Sequence in Peptide(s)	E-value
TLTWHTKTPVRP HFKHQHSYARPP AYSPISTVTQPY SHWWARVPFYPP	Replication protein A1(RPA1)	BAB09262.1	211	**WW**KI**I**R**FYP**	287
		AAC95163.1	219	**PISTV**	694
		NP_565571.1	273	**HFKH**	1250
		AAD48944.1	285	**WHTK**MW**PV**	215

DAMIMKKHWHRF	Geminivirus Rep interacting kinase 1 (GRIK1), GRIK2	NP_200863.2 NP_566876.3	164	**MIMK**	517

FPKAFHHHKIYK	Retinoblastoma like protein (pRBR)	BAB01449.1	317	**HKIY**	1250

SHEIYVGSDGFR	Anti silencing function 1b (ASF 1b), ASF1a	NP_198627.1 BAC54103.1	43	**IYVGS**	517

FHKHSPRSPIFI YALKHLPESTIP LLHAPYDHSVSP	RecQ Helicase, RecQ sim, RecQ4A	BAE98731.1	678	**FHK**S**SP**NTLAA**RS**A**I**	287
		BAE98731.1	326	**LKHLPS**I**I**	214
		NP_568499.1	697	**HAPYE**	932
		NP_172562.2	483	**LTYPLP**	694

TNVPNPLQPNPR GLLHHKHHRSPY	Werner Helicase - interacting protein	ABH03541.1	254	**NPLKPN**	694
		ABH03541.1	501	**LLHHK**	287

LITNNPGRLPPQ	RAD1	AAG42948.1	436	**ITNNP**	287
	RAD50	NP_565733.1	572	**GRLPPE**	386

QNNLDYIGLYAR TTNIYFNTPAEV	RAD5	NP_197667.1	42	**NI**I**FDTP**	694
		NP_197667.1	606	**QNNLEDLY**	663

SHEIYVGSDGFR CPLPYPLCLPHG	RAD4	NP_197166.2	556	**SHEIY**	160
		NP_197166.2	655	**PLCLP**	214

LEAPRPTPAVPM	RAD23-3	NP_974211.1	119	**AP**R**PTPA**	517
		NP_186903.1	129	**AP**A**PTR**PPP**PA**	31725

LITNNPGRLPPQ SHEIYVGSDGFR FHKEWRTHFQQR	RAD50	BAD94628.1	306	**KEWRT**H**FQQR**	160
		BAD94628.1	512	**HEIY**	932
		NP_565733.1	572	**GRLPPE**	386

The details of phage display identified peptides, proteins with the homologous regions, accession number of the proteins, starting co-ordinate of the matching region in the protein sequence, matching sequence in the phage peptide and the E-value of the corresponding match are shown. Residues in bold are identical (or similar in few cases) to the residues in the protein sequence. Mismatches to the protein sequence are shown in reduced font size.

**Table 4:** List of putative ToLCKeV AC3 interacting DNA and RNA polymerases

Peptide	Protein	Accession Number	Start	Interacting Sequence in Peptide(s)	E-value
WHQSWWAARLGQ	RNA dependant RNA polymerase (RDR1), RDR2	AAN64409.1 NP_192851.1	18	**AARLGQ**	160

LSPLYPQLLGLA	RDRP3, RDRP5	NP_179581.2	933	**LYPQALAL**	287

YPTSNIIPSIWS	RDR6	NP_190519.1	55	**YPNFEIADTSN**I**-PSI**	66
			1033	**DLIPEAW**	57117

HISPISAYPWVS	DNA pol γ2	NP_175498.2	17	**HLSP**S**SS--WVS**	694

HFKHQHSYARPP	DNA pol ε subunit	AAC77870.1	1855	**F**MD**QHNYA**	694

LITNNPGRLPPQ	DNA pol α subunit	AAG52305.1	115	**TN**KSQ**RLHP**	23644
			588	**NPGRL**	517

WHKHPHAVFNAR	DNA pol ζ catalytic subunit	AAG52299.1	1460	**H**R**IFNAR**	932

YALKHLPESTIP	DNA pol l	NP_172522.2	247	**LKHLP**	386

GPLLVLNSHSFD	DNA pol δ small subunit	NP_181742.2	311	**N**P**HSFD**	386

The details of phage display identified peptides, proteins with the homologous regions, accession number of the proteins, starting co-ordinate of the matching region in the protein sequence, matching sequence in the phage peptide and the E-value of the corresponding match are shown. Residues in bold are identical (or similar in few cases) to the residues in the protein sequence. Mismatches to the protein sequence are shown in reduced font size.

The proteins with at least two unique hits from different peptides and each with a minimum identity/similarity of five amino acids continuously or with one mismatch or gap were considered as putative interacting proteins. These interacting proteins were observed to belong to various metabolic and cellular processes, viz., transcription activation, cell cycle, kinases, replication, RNAi, histone and DNA modification (Tables 1,2,3, &4 and Additional file 1b). Identification of proteins from various cellular processes suggests that AC3 is likely to play role in these cellular processes. Since these putative interactions are only indicative, assays to investigate the impact of AC3 in these cellular processes is necessary for confirmation of its role.

### Construction of yeast vectors for analyzing the viral DNA replication

**Figure 1:**
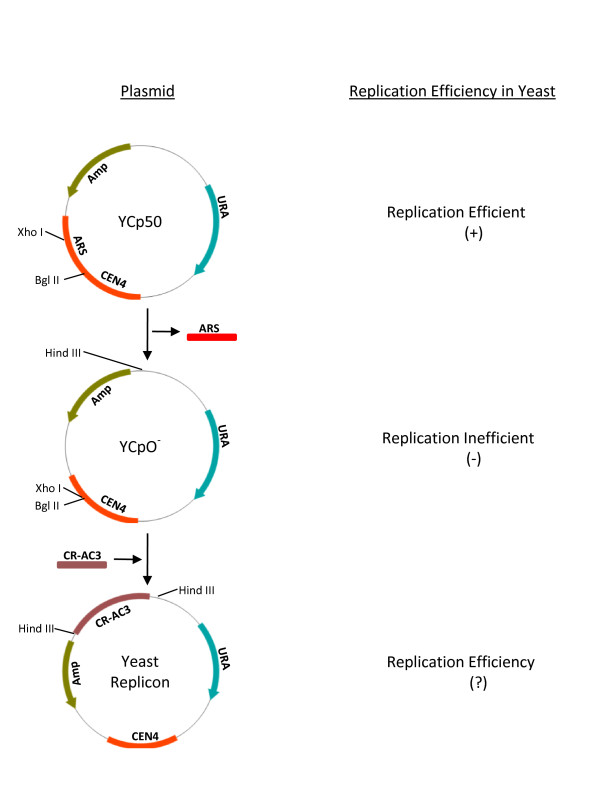
**Viral replicon construction in yeast**. Schematic diagram representing the construction of viral replicon in yeast. YCp50 is a binary plasmid that is capable of replication in bacteria and yeast. ARS and CEN4 sequences of the plasmid confer the ability to replicate in yeast. Removal of ARS fragment renders the plasmid unable to replicate in yeast (YCpO^-^). CR-AC3 fragment of the begomovirus contains the cis-acting sequences (origin of replication) and trans-acting viral genes (AC1, AC3) required for viral replication. Cloning of CR-AC3 of MYMIV at Hind III site was reported to confer YCpO^- ^the ability to replicate in yeast.

**Figure 2:**
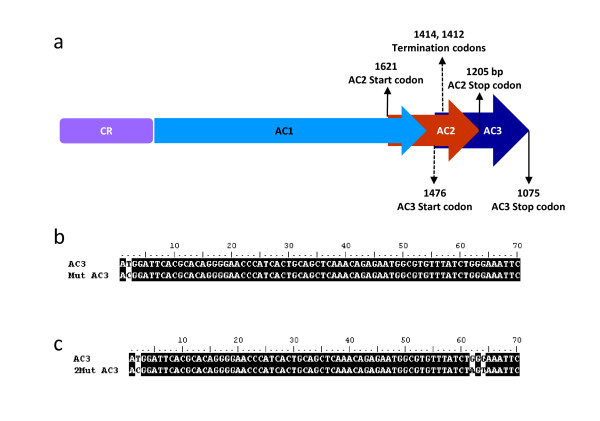
**Mutations in ToLCKeV AC3**. **(a) **Schematic diagram of the CR-AC3 region of the replicon constructs. The hyphenated arrows indicate the mutations in AC3 ORF. Numbers indicate the base positions with reference to the viral genome. **(b) **Sequence alignment of the AC3 mutated at start codon with the wild type AC3. The mutated base is shown against the white background at base number 2 in the AC3 ORF. Mutation was confirmed by sequencing. This mutant construct is denoted as CR-AC3^M^. **(c) **Termination mutations in AC3 were located at bases 62 and 64 of the AC3 ORF (denoted as CR-AC3^M21^). Details of these mutations have been explained in the text.

Budding yeast *S. cerevisiae *is known to support the replication of animal and plant RNA and DNA viruses including geminiviruses in the absence of complementing yeast autonomously replicating sequence (ARS) as an episome [70-72]. We have developed a vector system on the similar line of yeast vector developed for MYMIV [72]. The yeast vector YCp50 was modified to contain viral DNA spanning the entire viral origin of DNA replication (also called common region - CR or intergenic region - IR) region to AC3 (i.e., CR-AC3) replacing the ARS sequence (YCp-CRAC3) (Figure 1). This CR-AC3 region contains the complementary strand DNA with complete viral origin of DNA replication and viral ORFs AC1, AC2, AC3 and AC4. Another vector (YCp-CRAC3^M^) was constructed with a mutation (M1T) in the AC3 ORF that corresponds to the nucleotide change ATG to ACG (Figure 2). Such mutation would result only in a silent mutation in the overlapping AC2 ORF. We expected that this mutation would not produce any intact or N' terminal truncated AC3 protein since the second and only other methionine in AC3 protein is located at the C' terminus 133^rd ^amino acid position. Both the vectors YCp-CRAC3, YCp-CRAC3^M ^and the control YCp50 plasmids were transformed into yeast separately and the colony growth was monitored on selection medium (Ura^-^). Yeast transformed with YCp-CRAC3 and YCp-CRAC3^M ^exhibited much delayed growth phenotype (0.25-0.5 mm sized colonies in 5 days) in comparison to wild type plasmid YCp50 (3-4 mm size, Additional file 2). This kind of slow growth continued even after 10 days of incubation at standard conditions. This contrasted with the observation in case of MYMIV where the yeast was growing normally [72]. In our case, the delayed growth may be due to the possible toxicity of the viral proteins expressing in yeast. With this view further analyses were done *in planta*.

### Construction of plant vectors for analyzing the viral DNA replication

**Figure 3:**
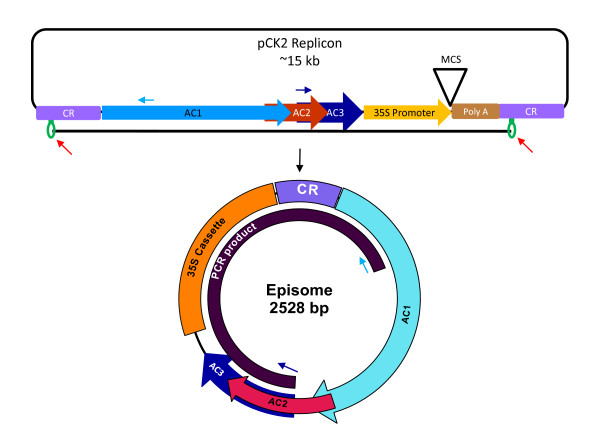
**Viral replicon used for *in planta *studies**. Viral replicon was constructed with the pCAMBIA1391Z binary vector. Complete replicon contains the region spanning from CR to AC3 (pCK2 replicon) and CR region of ToLCKeV. Presence of CR on either end in the same orientation enables the completion of rolling circle replication. Rolling circle replication releases the episome that contains only one complete CR and region spanning from AC1 to AC3. Red arrows indicate the nicking site of Rep protein in hairpin loop in either CRs and the black line represents the region of the vector that forms episome. Formation of an episome can be checked by PCR amplification with the oligonucleotides indicated by blue arrows. Internal primers were designed to amplify the DNA only from the episome under standardized PCR conditions. CR-AC3 is replaced by CR-AC3^M ^or CR-AC3^M21 ^for generating pCK2^M ^replicon and pCK2^M21 ^replicons. A 300 bp PCNA fragment was cloned into the MCS region to generate pCK2^M^-PCNA and pCK2^M21^-PCNA.

CR-AC3 region is reported to be sufficient to support viral replication in plants [17,73]. Since geminiviruses replicate by rolling circle replication by nicking and religating at the viral origin of DNA replication, we constructed vectors with viral origin of replication (CR) in the vector pCAMBIA1391Z. This vector was then modified to contain CR-AC3 or CR-AC3^M ^(AC3 mutated at start codon, Figure 2) in the same orientation as CR to generate pCK2 (Figure 3) and pCK2^M ^plasmids respectively. These vectors were used to agroinfiltrate in the tobacco leaves and the replication was observed at 4 dpi and 10 dpi. Time course analysis of the pCK2 and pCK2^M ^episome formation in tobacco plant leaves did not show any significant down-regulation in replication upon AC3 mutation (Data not shown). To rule out the reversion of the mutation in the start codon, we carried out sequencing of the episome and found that the mutation was preserved. Thus, the non-significant alteration in the replication efficiency might be due to various reasons: one being the minimal role of ToLCKeV AC3 in viral replication *in planta *unlike in protoplasts and leaf discs. It is also possible that the role of AC3 in viral replication occurs at a later stage requiring analysis of samples beyond 10 dpi. The other reason might be the permissiveness of the tobacco plant for the viral replication that masked the role of AC3. Such a conjecture gets support from an observation made in case of BCTV (California strain). When BCTV C3 was mutated, BCTV genome replicated to almost wild-type levels in tobacco plant whereas the replication was reduced in natural host plant sugar beet [74].

### ToLCKeV AC3 enhances viral replication in young tomato plants

To exclude the possibility of permissiveness of viral replication in tobacco, we performed an agroinoculation experiment with pCK2 and pCK2^M21 ^(with additional mutations in AC3 ORF) in the natural host tomato. Additional mutations in AC3 ORF corresponds to the amino acid positions 20 and 21 which are mutated to consecutive termination codons (Figure 2c). Since AC2 and AC3 ORFs overlap each other, we checked if these mutations have any effect on the AC2 protein sequence. While the mutation corresponding the 20^th ^amino acid in AC3 ORF is a silent mutation in AC2 ORF, the mutation in the 21^st ^amino acid of AC3 confers a change in the overlapping AC2 (G70V) ORF. Since 70^th ^amino acid of AC2 does not lie in any of the known functional domains (C'-terminal nuclear localisation signal, Zn finger motif and N'-terminus acidic transcription activation domain) required for silencing activity or transcriptional activation activity, we argued that such a mutation would not affect the functions of AC2.

**Figure 4:**
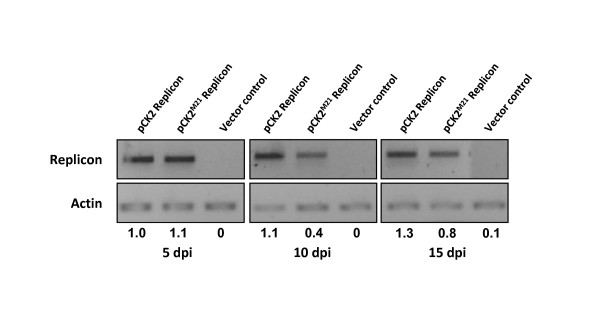
**Semi-quantitative amplification of episomal DNA from wild-type and AC3 mutated replicon**. Tomato plant leaves were infiltrated with wild-type replicon (pCK2) and AC3 mutated replicon (pCK2^M21^) separately. DNA from the infiltrated leaves was isolated at 5, 10 and 15 days post infiltration and subjected to Dpn I restriction digestion. Equal quantities of DNA were then used to amplify episome or actin. PCR conditions were specific to amplify only a part of replicon from the episome. Difference in the amplification of replicon in wild-type and mutant was prominent in the 10 dpi sample (3-4 folds difference). By 15 dpi, the difference in the amplification of episome was only 1.5-2 folds.

Examination of the relative replication levels of the episome between pCK2 replicon and pCK2^M21 ^replicon was carried out at various time intervals till 15 dpi (Figure 4). Within first five days, there was no difference in the levels of replication. However, the relative change in replication was more pronounced at 10 dpi as the replication of the wild-type replicon (pCK2) was 3-4 folds higher than that of AC3 mutant replicon (pCK2^M21^). The difference in the relative level of replication diminished to 1.5-2 folds at 15 dpi.

Our observation suggested that AC3 enhances replication but is not essential for replication. This is in line with earlier observation [18]. Role of AC3 was evident at 10-15 dpi. However, our results differed from published reports on the level of AC3 influence on viral replication. This might be due to the differences in the experimental design or the assay system. Earlier reports on AC3's role in replication were based on the analysis by mutating AC3 after the AC2 stop codon. This resulted in truncated AC3 with 80 amino acids in case of TGMV AC3 and more than 100 amino acids in other viruses [60,61,63-65,74]. In these studies it is possible that the truncation in the AC3 protein rendered it non-functional. It is also likely that the truncated AC3 interfered with the cellular pathways involved in replication. With its N'-terminus and middle region being intact, AC3 could titrate various proteins that interact with AC1 (like PCNA, pRBR, etc.). In such a case, the signal perceived by the N'-terminus of AC3 gets abruptly terminated being unable to relay the signal through a functional C'-terminus, thereby affecting replication. Our mutation strategy assured that AC3 is not expressed since we had mutated the start codon and included two stop codons at 20th and 21st amino acid positions. It is possible that in complete absence of AC3, another protein or an alternate pathway might rescue the viral replication [58]. This hypothesis gets considerable support from an experiment performed with transgenic plants. In their work Hayes et al. [75] raised various transgenic plants expressing DNA A ORFs and tandem repeats of DNA B genome. Plants expressing DNA A ORFs were crossed with transgenic plants containing DNA B as tandem repeats (2DNA B). When DNA from two such plants: AC1 × 2DNA B and AC1AC3 × 2DNA B were analyzed, the difference in the replication of DNA B in the presence and absence of AC3 was observed to be less than 1.5 fold indicating that the replication *in planta *was sustainable without AC3. Delay and amelioration of symptoms and reduced systemic movement of the virus in case of AC3 mutations observed *in planta *by agroinoculation experiments [18,60,63-65,74] suggest that AC3 has a more important role in systemic spread. Thus, the observed reduction in DNA levels at systemic locations is an indirect effect rather than its direct involvement in replication. Having a multitude of interacting partners that were discovered [12,14,59,76] and are being discovered, large multimer forming ability [66] that enables interaction with multiple partners indicate that AC3 is an important protein with multi-functional capability. Thus, further examination of its involvement in various cellular processes is needed.

The phage display data indicated that various ToLCKeV AC3 interacting peptides are homologous to the proteins of RNAi pathway. Interestingly, we found that few of these proteins (MOM1, MET1, DCL1, DCL2, AGO1, AGO2, AGO7, and HEN4) have multiple hits from different peptide sequences identified from phage display (Tables 1 and 2). We believed these proteins to be likely interacting partners of ToLCKeV-AC3. Hence, we investigated if AC3 could influence the RNAi pathway(s). One way to examine the role of AC3 in RNAi pathway is to study the silencing of an endogenous gene through the virus induced gene-silencing mechanism (VIGS) in the presence and absence of functional AC3 ORF.

### AC3 strongly manifests the phenotype associated with *PCNA *gene-silencing

**Figure 5:**
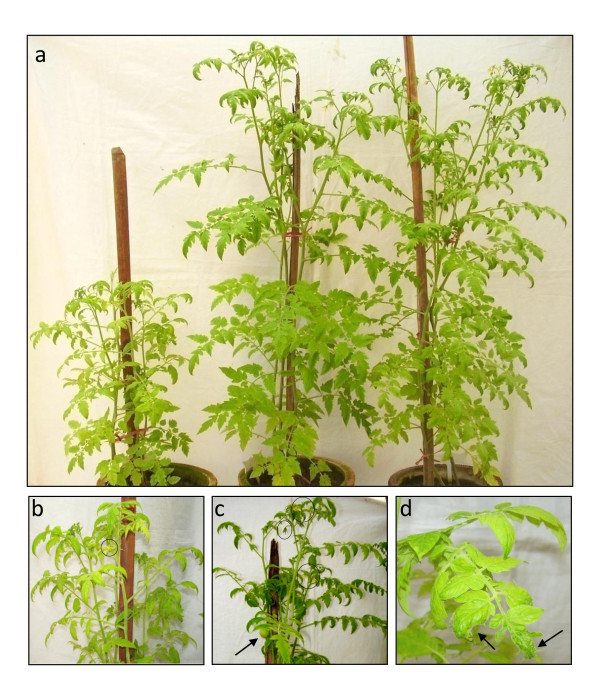
**Role of ToLCKeV AC3 on gene-silencing**. **(a) **High level transcription of a part of *PCNA *gene lead to the reduced growth of the plant. Retardation is observed in the growth of the plant agroinoculated with the wild type VIGS vector (wild type AC3) along with *PCNA *fragment under 35S promoter. Growth retardation is evident in this experiment by shortened height and decreased internodal distance between the stems of the tomato plant (plant on the left) and the plant agroinoculated with AC3 mutated VIGS vector (middle), control vector without any geminiviral DNA (right) and plant without any agroinoculation (not shown). **(b) **Growth retardation was coupled with reduced flowering (circle) and no fruits. **(c) **Normal flowering (circles) and developing fruits (arrow) were observed in plant agroinoculated with AC3 mutated VIGS vector. **(d) **Leaf morphology was altered in the plants agroinoculated with wild type VIGS vector.

CR-AC3 region for geminiviruses has been shown to be the minimal region required for eliciting VIGS [73]. Thus, we have utilized our pCK2 and pCK2^M21 ^replicon constructs to silence the endogenous gene *PCNA*. A 300 bp fragment of *PCNA *from tomato cDNA was cloned into the replicons (Figure 3). Agrobacterium containing one of the PCNA cloned replicons viz., pCK2-PCNA or pCK2^M21^-PCNA or control vector pC-PCNA were infiltrated into the leaves of tomato plants at 4 leaves stage. Growth of the plants was found to be normal and indistinguishable till 20 dpi. We noticed observable retardation in the growth of the pCK2-PCNA infiltrated plants at 30 dpi. By 45 dpi, the growth of the plants was severely stunted and was just half in length compared to plants infiltrated with pCK2^M21^-PCNA, pC-PCNA or uninfiltrated plants (Figure 5). Growth retardation was accompanied with reduced flowering, decreased internodal distance and absence of fruits at 45 dpi, whereas the formation of fruits and flowers were indistinguishable in plants infiltrated with pCK2^M21^-PCNA, pC-PCNA and plants without any infiltration (Table 5, Figure 5b-d). Retardation in growth of pCK2-PCNA infiltrated plants was relieved by 60 dpi which was evident by the rapid growth in the height of the infiltrated plants (data not shown).

**Table 5:** Plant height and inter-nodal distance of the tomato plants agroinfiltrated with VIGS vectors at 45 dpi

	Vector Infiltrated			
	
	pCK2-PCNA	pCK2M21-PCNA	pC-PCNA	No Vector
**Number of Plants**	10	10	10	10
**Average Height (in cm)**	64	105	102	110
**Number of Internodes**	12	11	11	12
**Average Internodal Distance (in cm)**	5.33	9.54	9.27	9.16

*PCNA *gene is required for the replication of DNA. It is expressed in meristematic tissues that rapidly divide and grow. PCNA is absent in the mature leaves [77]. So, silencing of endogenous *PCNA *would hamper the DNA replication in the rapidly growing tissues resulting in stunted growth - an easily recognisable phenotype [78,79]. In our case plant growth was severely retarded which was evident from the reduced plant height, flowering and absence of fruits. Another advantage of our VIGS construct is the absence of virion sense strand ORFs AV1 and AV2. Absence of these proteins prevents virion packaging and movement of virion particle. So, by design, our VIGS vector is movement defective and cannot produce disease symptoms [73,80]. Thus, the observed deformities in the plant growth are due to the silencing of *PCNA*.

Growth retardation observed in our experiments in the presence of AC3 indicates that AC3 could have strong influence on virus induced gene-silencing of endogenous gene *PCNA* in this experiment. However, it is difficult to ascertain the exact role of AC3 in RNAi and with which proteins it actually interacts from our experiment in isolation.

## Conclusions

In this study we have identified various ToLCKeV AC3 interacting peptides through phage display analysis. Few of these interacting peptides were found to be homologous to proteins from replication process, RNAi pathway, histone and DNA modifying enzymes indicating the role of AC3 in these pathways. In order to verify if ToLCKeV AC3 has any role in any of these metabolisms, we have developed vectors to investigate its role in replication and gene-silencing. We observed that ToLCKeV AC3 effectively functions in the viral replication at an intermediate stage and enhances replication in host plant tomato. In the gene-silencing mechanism, the phenotype associated with the host gene *PCNA *silencing was strongly manifested in the presence of functional AC3 ORF. These observations indicate that the role of AC3 extends to RNAi pathway in addition to its role in DNA replication.

## Methods

### Phage Display analysis

We have used the 'Ph.D-12 phage display library' kit (New England Biolabs) for analyzing the various peptides that interact with AC3 protein. The protocol was followed as per the technical bulletin of the kit. In brief, the panning was carried out by incubating a library of phage-displayed peptides with a plate coated with the purified MBP-AC3 or MBP [66] in the TBST (100 mM Tris-HCl, 150 mM NaCl, pH 7.5, 0.1% Tween20) binding buffer (1.5 × 10^11 ^phage diluted in 1ml buffer). Unbound phages were removed by washing with TBST. Bound phages were eluted with elution buffer (0.2 M Glycine-HCl, pH 2.2; 1 mg/ml BSA) and neutralized with 1 M Tris-HCl (pH 9.1). The eluted phages were then amplified with *E. coli *ER2738 bacterial strain. Amplified phages were then subjected to two more rounds of panning and taken through additional binding/amplification cycles to enrich the pool in favor of binding sequences. After three rounds, individual clones were characterized by DNA sequencing. Exclusive phage sequences were obtained after removing the M13 phage sequences. These DNA sequences were translated as per the reduced genetic code for M13 phage in *E. coli *ER2738. The sequence of the peptides was analyzed by 'BioEdit' software and the peptides common in MBP-AC3 and MBP interacting peptides were removed. Each peptide sequence thus obtained was then searched for homologous peptide sequences in proteins against the *Arabidopsis *non-redundant protein database at NCBI through 'blastp' programme adjusted for small sequence analysis. Initially we have searched for the known AC3 interacting proteins in the blast hits and have taken the E-value of pRBR (blast hit observed for the peptide sequence "FPKAFHHHKIYK" as the threshold for filtering the blast results. Further, we have shortlisted only those proteins with at least two hits from the same or different peptides or those with E-value less than the blast hit of GRIK1, another protein known to interact with Rep.

### Site directed mutagenesis

AC3 ORF was mutated at three sites - one at base position 2 and others at bases 62 and 64 of AC3 ORF in two steps. Initially, the first mutation was carried out at the second base of AC3 ORF with overlapping oligos for both strands (AC^M ^Fwd and AC^M ^Rev). These oligos were used to amplify the whole pGEMT-Easy vector containing the wild type CR-AC3 region of the virus. The resulting amplified vector was incubated with T4 polynucleotide kinase (MBI Fermentas) along with T4- DNA ligase (MBI Fermentas) in the ligation reaction mix. The ligated products were transformed into *E. coli *DH5α. Plasmids were isolated from each bacterial colony and sequenced to confirm the site-directed mutation. This plasmid containing mutated AC3 ORF at start codon (CR-AC3^M^) was utilized to generate two more site-directed mutations at bases 62 and 64 with the oligos AC3^M21 ^Fwd, AC3^M21 ^Rev. The resulting construct was named CR-AC3^M21^. Sequence of the oligos used was:

AC3^M ^Fwd: 5'- GTTCTGCAACGTGCACGGATTCACGCACAGG-3'

AC3^M ^Rev: 5'- CCTGTGCGTGAATCCGTGCACGTTGCAGAAC-3'

AC3^M21 ^Fwd: 5'- GGCGTGTTTATCTAGTAAATTCAAAATCCC-3'

AC3^M21 ^Rev: 5'- GGGATTTTGAATTTACTAGATAAACACGCC-3'

### Construction of yeast replicons

ARS containing yeast plasmid YCp50 was subjected to restriction digestion with Xho I and Bgl II to delete part of the ARS sequence rendering it replication deficient. The resulting plasmid is ligated by end filling and is called YCpO^-^. pGEMT-Easy clones containing CR-AC3 or CR-AC3^M ^region were digested with Hind III restriction enzyme. The resulting CR-AC3 and CR-AC3^M ^were cloned into Hind III site of YCpO^- ^to generate YCp-CRAC3 or YCp-CRAC3^M^.

### Construction of plant replicons and VIGS vectors

Hind III and EcoR I digested CaMV 35S cassette from pBI121 plasmid was cloned into Hind III and EcoR I digested plant binary vector pCAMBIA1391Z. EcoR I digested CR region of the ToLCKeV genome was cloned adjacent to the 35S cassette to generate pC. CR-AC3 or CR-AC3^M21 ^was cloned into the Hind III site of the pC vector to generate pCK2 or pCK2^M21 ^respectively. These plasmids were transformed into *Agrobacterium tumefaciens *(LBA4404). Cultures from single colonies of agrobacterium were grown and used for agroinfiltration studies. VIGS vectors were constructed by cloning 300 bp tomato PCNA into the BamH I site of the pCK2 or pCK2^M21^. Oligos used to amplify the PCNA fragment were:

PCNA Fwd: 5'- ACGGATCCGTTCTAGAATCGATTAAGGATCTGG- 3'

PCNA Rev: 5'- GGGGATCCCCATTAGCTTCATCTCAAAATCAG- 3'

### 3.3.13.2 Transient replication assay in plant leaves

The binary plasmid containing pCK2 replicon or pCK2^M21 ^replicon containing agrobacterium was grown in YEM at 30°C till OD_600 _≈ 1.0-2.0. Cells were harvested and washed with sterile YEM to remove antibiotic. Agrobacterium cells were resuspended in YEM to an OD_600 _≈ 1.0-2.0 and then agroinfiltrated by injecting into tobacco or tomato leaves. Infiltrated leaves were collected at various intervals (5, 10, 15 days post inoculation) and genomic DNA was extracted. This genomic DNA was subjected to Dpn I treatment to remove the episomal DNA originated from agrobacterium. To quantitate the episomal DNA, PCR was done with following divergent primers (AC^M ^Fwd, AC1 Rev119) and the amplification was visualized through agarose gel electrophoresis. *Actin *amplification (using Actin Fwd, Actin Rev oligos) was used as control.

AC3^M ^Fwd: 5'- GTTCTGCAACGTGCACGGATTCACGCACAGG-3'

AC1 Rev119: 5'- AGCTCGAGCTAATCGACTTGGAAAAC-3'

Actin Fwd: 5'- ATGCCATTCTCCGTCTTGACTTG-3'

Actin Rev: 5'- GAGTTGTATGTAGTCTCGTGGATT-3'

## Competing interests

The authors declare that they have no competing interests.

## Authors' contributions

KKP had done all the experiments and drafted the manuscript. KKP, SKM and NRC together designed the experiments. SKM and NRC had proof-read and finalized the manuscript. All the authors read and approved the final manuscript.

## Supplementary Material

Additional file 1 **List of ToLCKeV AC3 interacting phage peptides and putative interacting proteins**. Representative peptides that are interacting with AC3 are shown in additional file 1a. Proteins that contain at least five contiguous amino acids identical to the 12mer peptide obtained from phage display are listed in additional file 1b. Click here for file

Additional file 2 **Replication efficiency of ToLCKeV in yeast**. Yeast cells were transformed with wild type YCp50 plasmid or YCp-CRAC3 (ToLCKeV) and incubated at 30°C for five days in Ura^- ^medium. Yeast transformed with YCp50 grew normally while yeast transformed with YCp-CRAC3 (ToLCKeV) exhibited delayed growth. Click here for file

## References

[B1] Goodman RM Single-stranded DNA genome in a whitefly-transmitted plant virus Virology 1977 83 171 179 10.1016/0042-6822(77)90220-3 18625485

[B2] Kheyr-Pour A Bendahmane M Matzeit V Accotto GP Crespi S Gronenborn B Tomato yellow leaf curl virus from Sardinia is a whitefly-transmitted monopartite geminivirus Nucleic Acids Res 1991 19 6763 6769 10.1093/nar/19.24.6763 1840676 PMC329307

[B3] Dry IB Krake LR Rigden JE Rezaian MA A novel subviral agent associated with a geminivirus: the first report of a DNA satellite Proc Natl Acad Sci USA 1997 94 7088 7093 10.1073/pnas.94.13.7088 9192696 PMC21289

[B4] Hamilton WD Bisaro DM Buck KW Identification of novel DNA forms in tomato golden mosaic virus infected tissue. Evidence for a two component viral genome Nucleic Acids Res 1982 10 4901 4912 10.1093/nar/10.16.4901 6290994 PMC320840

[B5] Hamilton WD Bisaro DM Coutts RH Buck KW Demonstration of the bipartite nature of the genome of a single-stranded DNA plant virus by infection with the cloned DNA components Nucleic Acids Res 1983 11 7387 7396 10.1093/nar/11.21.7387 6417624 PMC326490

[B6] Rogers SG Bisaro DM Horsch RB Fraley RT Hoffmann NL Brand L Elmer JS Lloyd AM Tomato golden mosaic virus A component DNA replicates autonomously in transgenic plants Cell 1986 45 593 600 10.1016/0092-8674(86)90291-6 3708687

[B7] Donson J Gunn HV Woolston CJ Pinner MS Boulton MI Mullineaux PM Davies JW Agrobacterium-mediated infectivity of cloned digitaria streak virus DNA Virology 1988 162 248 250 10.1016/0042-6822(88)90416-3 3341112

[B8] Nagar S Pedersen TJ Carrick KM Hanley-Bowdoin L Robertson D A geminivirus induces expression of a host DNA synthesis protein in terminally differentiated plant cells Plant Cell 1995 7 705 719 10.1105/tpc.7.6.705 7647562 PMC160820

[B9] Kong LJ Orozco BM Roe JL Nagar S Ou S Feiler HS Durfee T Miller AB Gruissem W Robertson D Hanley-Bowdoin L A geminivirus replication protein interacts with the retinoblastoma protein through a novel domain to determine symptoms and tissue specificity of infection in plants Embo J 2000 19 3485 3495 10.1093/emboj/19.13.3485 10880461 PMC313951

[B10] Bass HW Nagar S Hanley-Bowdoin L Robertson D Chromosome condensation induced by geminivirus infection of mature plant cells J Cell Sci 2000 113 (Pt 7) 1149 1160 10704366 10.1242/jcs.113.7.1149

[B11] Nagar S Hanley-Bowdoin L Robertson D Host DNA replication is induced by geminivirus infection of differentiated plant cells Plant Cell 2002 14 2995 3007 10.1105/tpc.005777 12468723 PMC151198

[B12] Settlage SB Miller AB Gruissem W Hanley-Bowdoin L Dual interaction of a geminivirus replication accessory factor with a viral replication protein and a plant cell cycle regulator Virology 2001 279 570 576 10.1006/viro.2000.0719 11162812

[B13] Luque A Sanz-Burgos AP Ramirez-Parra E Castellano MM Gutierrez C Interaction of geminivirus Rep protein with replication factor C and its potential role during geminivirus DNA replication Virology 2002 302 83 94 10.1006/viro.2002.1599 12429518

[B14] Castillo AG Collinet D Deret S Kashoggi A Bejarano ER Dual interaction of plant PCNA with geminivirus replication accessory protein (Ren) and viral replication protein (Rep) Virology 2003 312 381 394 10.1016/S0042-6822(03)00234-4 12919743

[B15] Castillo AG Kong LJ Hanley-Bowdoin L Bejarano ER Interaction between a geminivirus replication protein and the plant sumoylation system J Virol 2004 78 2758 2769 10.1128/JVI.78.6.2758-2769.2004 14990696 PMC353736

[B16] Bagewadi B Chen S Lal SK Choudhury NR Mukherjee SK PCNA interacts with Indian mung bean yellow mosaic virus rep and downregulates Rep activity J Virol 2004 78 11890 11903 10.1128/JVI.78.21.11890-11903.2004 15479830 PMC523298

[B17] Singh DK Islam MN Choudhury NR Karjee S Mukherjee SK The 32 kDa subunit of replication protein A (RPA) participates in the DNA replication of Mung bean yellow mosaic India virus (MYMIV) by interacting with the viral Rep protein Nucleic Acids Res 2007 35 755 770 10.1093/nar/gkl1088 17182628 PMC1807949

[B18] Elmer JS Brand L Sunter G Gardiner WE Bisaro DM Rogers SG Genetic analysis of the tomato golden mosaic virus. II. The product of the AL1 coding sequence is required for replication Nucleic Acids Res 1988 16 7043 7060 10.1093/nar/16.14.7043 3405758 PMC338350

[B19] Gorbalenya AE Koonin EV Donchenko AP Blinov VM A novel superfamily of nucleoside triphosphate-binding motif containing proteins which are probably involved in duplex unwinding in DNA and RNA replication and recombination FEBS Lett 1988 235 16 24 10.1016/0014-5793(88)81226-2 2841153 PMC7130140

[B20] Fontes EP Luckow VA Hanley-Bowdoin L A geminivirus replication protein is a sequence-specific DNA binding protein Plant Cell 1992 4 597 608 10.1105/tpc.4.5.597 1498611 PMC160156

[B21] Lazarowitz SG Wu LC Rogers SG Elmer JS Sequence-specific interaction with the viral AL1 protein identifies a geminivirus DNA replication origin Plant Cell 1992 4 799 809 10.1105/tpc.4.7.799 1392596 PMC160175

[B22] Gladfelter HJ Eagle PA Fontes EP Batts L Hanley-Bowdoin L Two domains of the AL1 protein mediate geminivirus origin recognition Virology 1997 239 186 197 10.1006/viro.1997.8869 9426458

[B23] Higashitani A Greenstein D Hirokawa H Asano S Horiuchi K Multiple DNA conformational changes induced by an initiator protein precede the nicking reaction in a rolling circle replication origin J Mol Biol 1994 237 388 400 10.1006/jmbi.1994.1242 8151700

[B24] Laufs J Schumacher S Geisler N Jupin I Gronenborn B Identification of the nicking tyrosine of geminivirus Rep protein FEBS Lett 1995 377 258 262 10.1016/0014-5793(95)01355-5 8543063

[B25] Hafner GJ Stafford MR Wolter LC Harding RM Dale JL Nicking and joining activity of banana bunchy top virus replication protein in vitro J Gen Virol 1997 78 Pt 7 1795 1799 9225058 10.1099/0022-1317-78-7-1795

[B26] Pant V Gupta D Choudhury NR Malathi VG Varma A Mukherjee SK Molecular characterization of the Rep protein of the blackgram isolate of Indian mungbean yellow mosaic virus J Gen Virol 2001 82 2559 2567 11562548 10.1099/0022-1317-82-10-2559

[B27] Laufs J Traut W Heyraud F Matzeit V Rogers SG Schell J Gronenborn B In vitro cleavage and joining at the viral origin of replication by the replication initiator protein of tomato yellow leaf curl virus Proc Natl Acad Sci USA 1995 92 3879 3883 10.1073/pnas.92.9.3879 7732000 PMC42065

[B28] Sunter G Hartitz MD Bisaro DM Tomato golden mosaic virus leftward gene expression: autoregulation of geminivirus replication protein Virology 1993 195 275 280 10.1006/viro.1993.1374 8317105

[B29] Groning BR Hayes RJ Buck KW Simultaneous regulation of tomato golden mosaic virus coat protein and AL1 gene expression: expression of the AL4 gene may contribute to suppression of the AL1 gene J Gen Virol 1994 75 (Pt 4) 721 726 10.1099/0022-1317-75-4-721 8151290

[B30] Shung CY Sunter G AL1-dependent repression of transcription enhances expression of Tomato golden mosaic virus AL2 and AL3 Virology 2007 364 112 122 10.1016/j.virol.2007.03.006 17407785 PMC2902176

[B31] Desbiez C David C Mettouchi A Laufs J Gronenborn B Rep protein of tomato yellow leaf curl geminivirus has an ATPase activity required for viral DNA replication Proc Natl Acad Sci USA 1995 92 5640 5644 10.1073/pnas.92.12.5640 7777563 PMC41752

[B32] Choudhury NR Malik PS Singh DK Islam MN Kaliappan K Mukherjee SK The oligomeric Rep protein of Mungbean yellow mosaic India virus (MYMIV) is a likely replicative helicase Nucleic Acids Res 2006 34 6362 6377 10.1093/nar/gkl903 17142233 PMC1669733

[B33] Clerot D Bernardi F DNA helicase activity is associated with the replication initiator protein rep of tomato yellow leaf curl geminivirus J Virol 2006 80 11322 11330 10.1128/JVI.00924-06 16943286 PMC1642161

[B34] Kong LJ Hanley-Bowdoin L A geminivirus replication protein interacts with a protein kinase and a motor protein that display different expression patterns during plant development and infection Plant Cell 2002 14 1817 1832 10.1105/tpc.003681 12172024 PMC151467

[B35] Arguello-Astorga G Lopez-Ochoa L Kong LJ Orozco BM Settlage SB Hanley-Bowdoin L A novel motif in geminivirus replication proteins interacts with the plant retinoblastoma-related protein J Virol 2004 78 4817 4826 10.1128/JVI.78.9.4817-4826.2004 15078963 PMC387707

[B36] Settlage SB Miller AB Hanley-Bowdoin L Interactions between geminivirus replication proteins J Virol 1996 70 6790 6795 8794317 10.1128/jvi.70.10.6790-6795.1996 PMC190723

[B37] Malik PS Kumar V Bagewadi B Mukherjee SK Interaction between coat protein and replication initiation protein of Mung bean yellow mosaic India virus might lead to control of viral DNA replication Virology 2005 337 273 283 10.1016/j.virol.2005.04.030 15913696

[B38] Hartitz MD Sunter G Bisaro DM The tomato golden mosaic virus transactivator (TrAP) is a single-stranded DNA and zinc-binding phosphoprotein with an acidic activation domain Virology 1999 263 1 14 10.1006/viro.1999.9925 10544077

[B39] Ruiz-Medrano R Guevara-Gonzalez RG Arguello-Astorga GR Monsalve-Fonnegra Z Herrera-Estrella LR Rivera-Bustamante RF Identification of a sequence element involved in AC2-mediated transactivation of the pepper huasteco virus coat protein gene Virology 1999 253 162 169 10.1006/viro.1998.9484 9918875

[B40] Sunter G Bisaro DM Identification of a minimal sequence required for activation of the tomato golden mosaic virus coat protein promoter in protoplasts Virology 2003 305 452 462 10.1006/viro.2002.1757 12573590

[B41] Sunter G Bisaro DM Regulation of a geminivirus coat protein promoter by AL2 protein (TrAP): evidence for activation and derepression mechanisms Virology 1997 232 269 280 10.1006/viro.1997.8549 9191840

[B42] Voinnet O Pinto YM Baulcombe DC Suppression of gene silencing: a general strategy used by diverse DNA and RNA viruses of plants Proc Natl Acad Sci USA 1999 96 14147 14152 10.1073/pnas.96.24.14147 10570213 PMC24205

[B43] Van Wezel R Liu H Wu Z Stanley J Hong Y Contribution of the zinc finger to zinc and DNA binding by a suppressor of posttranscriptional gene silencing J Virol 2003 77 696 700 10.1128/JVI.77.1.696-700.2003 12477872 PMC140617

[B44] Trinks D Rajeswaran R Shivaprasad PV Akbergenov R Oakeley EJ Veluthambi K Hohn T Pooggin MM Suppression of RNA silencing by a geminivirus nuclear protein, AC2, correlates with transactivation of host genes J Virol 2005 79 2517 2527 10.1128/JVI.79.4.2517-2527.2005 15681452 PMC546592

[B45] Wang H Hao L Shung CY Sunter G Bisaro DM Adenosine kinase is inactivated by geminivirus AL2 and L2 proteins Plant Cell 2003 15 3020 3032 10.1105/tpc.015180 14615595 PMC282852

[B46] Wang H Buckley KJ Yang X Buchmann RC Bisaro DM Adenosine kinase inhibition and suppression of RNA silencing by geminivirus AL2 and L2 proteins J Virol 2005 79 7410 7418 10.1128/JVI.79.12.7410-7418.2005 15919897 PMC1143688

[B47] Buchmann RC Asad S Wolf JN Mohannath G Bisaro DM Geminivirus AL2 and L2 proteins suppress transcriptional gene silencing and cause genome-wide reductions in cytosine methylation J Virol 2009 83 5005 5013 10.1128/JVI.01771-08 19279102 PMC2682068

[B48] Hao L Wang H Sunter G Bisaro DM Geminivirus AL2 and L2 proteins interact with and inactivate SNF1 kinase Plant Cell 2003 15 1034 1048 10.1105/tpc.009530 12671096 PMC152347

[B49] Vanitharani R Chellappan P Pita JS Fauquet CM Differential roles of AC2 and AC4 of cassava geminiviruses in mediating synergism and suppression of posttranscriptional gene silencing J Virol 2004 78 9487 9498 10.1128/JVI.78.17.9487-9498.2004 15308741 PMC506916

[B50] Gopal P Pravin Kumar P Sinilal B Jose J Kasin Yadunandam A Usha R Differential roles of C4 and betaC1 in mediating suppression of post-transcriptional gene silencing: evidence for transactivation by the C2 of Bhendi yellow vein mosaic virus, a monopartite begomovirus Virus Res 2007 123 9 18 10.1016/j.virusres.2006.07.014 16949698

[B51] Rojas MR Jiang H Salati R Xoconostle-Cazares B Sudarshana MR Lucas WJ Gilbertson RL Functional analysis of proteins involved in movement of the monopartite begomovirus, Tomato yellow leaf curl virus Virology 2001 291 110 125 10.1006/viro.2001.1194 11878881

[B52] Jupin I De Kouchkovsky F Jouanneau F Gronenborn B Movement of tomato yellow leaf curl geminivirus (TYLCV): involvement of the protein encoded by ORF C4 Virology 1994 204 82 90 10.1006/viro.1994.1512 8091687

[B53] Latham JR Saunders K Pinner MS Stanley J Induction of plant cell division by beet curly top virus gene C4 The Plant Journal 1997 11 1273 1283 10.1046/j.1365-313X.1997.11061273.x

[B54] Eagle PA Hanley-Bowdoin L cis elements that contribute to geminivirus transcriptional regulation and the efficiency of DNA replication J Virol 1997 71 6947 6955 9261423 10.1128/jvi.71.9.6947-6955.1997 PMC191979

[B55] Piroux N Saunders K Page A Stanley J Geminivirus pathogenicity protein C4 interacts with Arabidopsis thaliana shaggy-related protein kinase AtSKeta, a component of the brassinosteroid signalling pathway Virology 2007 362 428 440 10.1016/j.virol.2006.12.034 17280695

[B56] Xie Q Suarez-Lopez P Gutierrez C Identification and analysis of a retinoblastoma binding motif in the replication protein of a plant DNA virus: requirement for efficient viral DNA replication Embo J 1995 14 4073 4082 7664747 10.1002/j.1460-2075.1995.tb00079.x PMC394486

[B57] Yang X Baliji S Buchmann RC Wang H Lindbo JA Sunter G Bisaro DM Functional modulation of the geminivirus AL2 transcription factor and silencing suppressor by self-interaction J Virol 2007 81 11972 11981 10.1128/JVI.00617-07 17715241 PMC2168806

[B58] Settlage SB See RG Hanley-Bowdoin L Geminivirus C3 protein: replication enhancement and protein interactions J Virol 2005 79 9885 9895 10.1128/JVI.79.15.9885-9895.2005 16014949 PMC1181577

[B59] Selth LA Dogra SC Rasheed MS Healy H Randles JW Rezaian MA A NAC domain protein interacts with tomato leaf curl virus replication accessory protein and enhances viral replication Plant Cell 2005 17 311 325 10.1105/tpc.104.027235 15608335 PMC544507

[B60] Sung YK Coutts RH Mutational analysis of potato yellow mosaic geminivirus J Gen Virol 1995 76 Pt 7 1773 1780 10.1099/0022-1317-76-7-1773 9049382

[B61] Sunter G Hartitz MD Hormuzdi SG Brough CL Bisaro DM Genetic analysis of tomato golden mosaic virus: ORF AL2 is required for coat protein accumulation while ORF AL3 is necessary for efficient DNA replication Virology 1990 179 69 77 10.1016/0042-6822(90)90275-V 2219741

[B62] Sunter G Stenger DC Bisaro DM Heterologous complementation by geminivirus AL2 and AL3 genes Virology 1994 203 203 210 10.1006/viro.1994.1477 8053144

[B63] Hormuzdi SG Bisaro DM Genetic analysis of beet curly top virus: examination of the roles of L2 and L3 genes in viral pathogenesis Virology 1995 206 1044 1054 10.1006/viro.1995.1027 7856079

[B64] Etessami P Saunders K Watts J Stanley J Mutational analysis of complementary-sense genes of African cassava mosaic virus DNA A J Gen Virol 1991 72 Pt 5 1005 1012 10.1099/0022-1317-72-5-1005 2033385

[B65] Morris B Richardson K Eddy P Zhan XC Haley A Gardner R Mutagenesis of the AC3 open reading frame of African cassava mosaic virus DNA A reduces DNA B replication and ameliorates disease symptoms J Gen Virol 1991 72 (Pt 6) 1205 1213 10.1099/0022-1317-72-6-1205 2045787

[B66] Pasumarthy K Choudhury N Mukherjee S Tomato leaf curl Kerala virus (ToLCKeV) AC3 protein forms a higher order oligomer and enhances ATPase activity of replication initiator protein (Rep/AC1) Virol J 2010 7 128 10.1186/1743-422X-7-128 20546567 PMC2901266

[B67] Pedersen TJ Hanley-Bowdoin L Molecular characterization of the AL3 protein encoded by a bipartite geminivirus Virology 1994 202 1070 1075 10.1006/viro.1994.1442 8030214

[B68] Obenauer JC Cantley LC Yaffe MB Scansite 2.0: Proteome-wide prediction of cell signaling interactions using short sequence motifs Nucleic Acids Res 2003 31 3635 3641 10.1093/nar/gkg584 12824383 PMC168990

[B69] Shen W Hanley-Bowdoin L Geminivirus infection up-regulates the expression of two Arabidopsis protein kinases related to yeast SNF1- and mammalian AMPK-activating kinases Plant Physiol 2006 142 1642 1655 10.1104/pp.106.088476 17041027 PMC1676070

[B70] Janda M Ahlquist P Brome mosaic virus RNA replication protein 1a dramatically increases in vivo stability but not translation of viral genomic RNA3 Proc Natl Acad Sci USA 1998 95 2227 2232 10.1073/pnas.95.5.2227 9482867 PMC19301

[B71] Angeletti PC Kim K Fernandes FJ Lambert PF Stable replication of papillomavirus genomes in Saccharomyces cerevisiae J Virol 2002 76 3350 3358 10.1128/JVI.76.7.3350-3358.2002 11884560 PMC136042

[B72] Raghavan V Malik PS Choudhury NR Mukherjee SK The DNA-A component of a plant geminivirus (Indian mung bean yellow mosaic virus) replicates in budding yeast cells J Virol 2004 78 2405 2413 10.1128/JVI.78.5.2405-2413.2004 14963136 PMC369238

[B73] Pandey P Choudhury NR Mukherjee SK A geminiviral amplicon (VA) derived from Tomato leaf curl virus (ToLCV) can replicate in a wide variety of plant species and also acts as a VIGS vector Virol J 2009 6 152 10.1186/1743-422X-6-152 19788728 PMC2761890

[B74] Stanley J Latham JR Pinner MS Bedford I Markham PG Mutational analysis of the monopartite geminivirus beet curly top virus Virology 1992 191 396 405 10.1016/0042-6822(92)90201-Y 1413511

[B75] Hayes RJ Buck KW Replication of tomato golden mosaic virus DNA B in transgenic plants expressing open reading frames (ORFs) of DNA A: requirement of ORF AL2 for production of single-stranded DNA Nucleic Acids Res 1989 17 10213 10222 10.1093/nar/17.24.10213 2602150 PMC335295

[B76] Gutierrez C Geminivirus DNA replication Cell Mol Life Sci 1999 56 313 329 10.1007/s000180050433 11212359 PMC11146802

[B77] Kelman Z PCNA: structure, functions and interactions Oncogene 1997 14 629 640 10.1038/sj.onc.1200886 9038370

[B78] Peele C Jordan CV Muangsan N Turnage M Egelkrout E Eagle P Hanley-Bowdoin L Robertson D Silencing of a meristematic gene using geminivirus-derived vectors Plant J 2001 27 357 366 10.1046/j.1365-313x.2001.01080.x 11532181

[B79] Kjemtrup S Sampson KS Peele CG Nguyen LV Conkling MA Thompson WF Robertson D Gene silencing from plant DNA carried by a Geminivirus Plant J 1998 14 91 100 10.1046/j.1365-313X.1998.00101.x 15494056

[B80] Huang Z Qiang C Brooke H Charles A Hugh M A DNA replicon system for rapid high-level production of virus-like particles in plants Biotechnology and Bioengineering 2009 103 706 714 10.1002/bit.22299 19309755 PMC2704498

