# Supplementary material for: The presence of tomato leaf curl Kerala virus AC3 protein enhances viral DNA replication and modulates virus induced gene-silencing mechanism in tomato plants
Source: Virol J. 2011 Apr 18;8:178. doi: 10.1186/1743-422X-8-178 (PMC3102638; doi:10.1186/1743-422X-8-178)
Supplement: Additional file 1 — List of ToLCKeV AC3 interacting phage peptides and putative interacting proteins. Representative peptides that are interacting with AC3 are shown in additional file 1a. Proteins that contain at least five contiguous amino acids identical to the 12mer peptide obtained from phage display are listed in additional file 1b. [file 1743-422X-8-178-S1.PPT]

## Slide 1
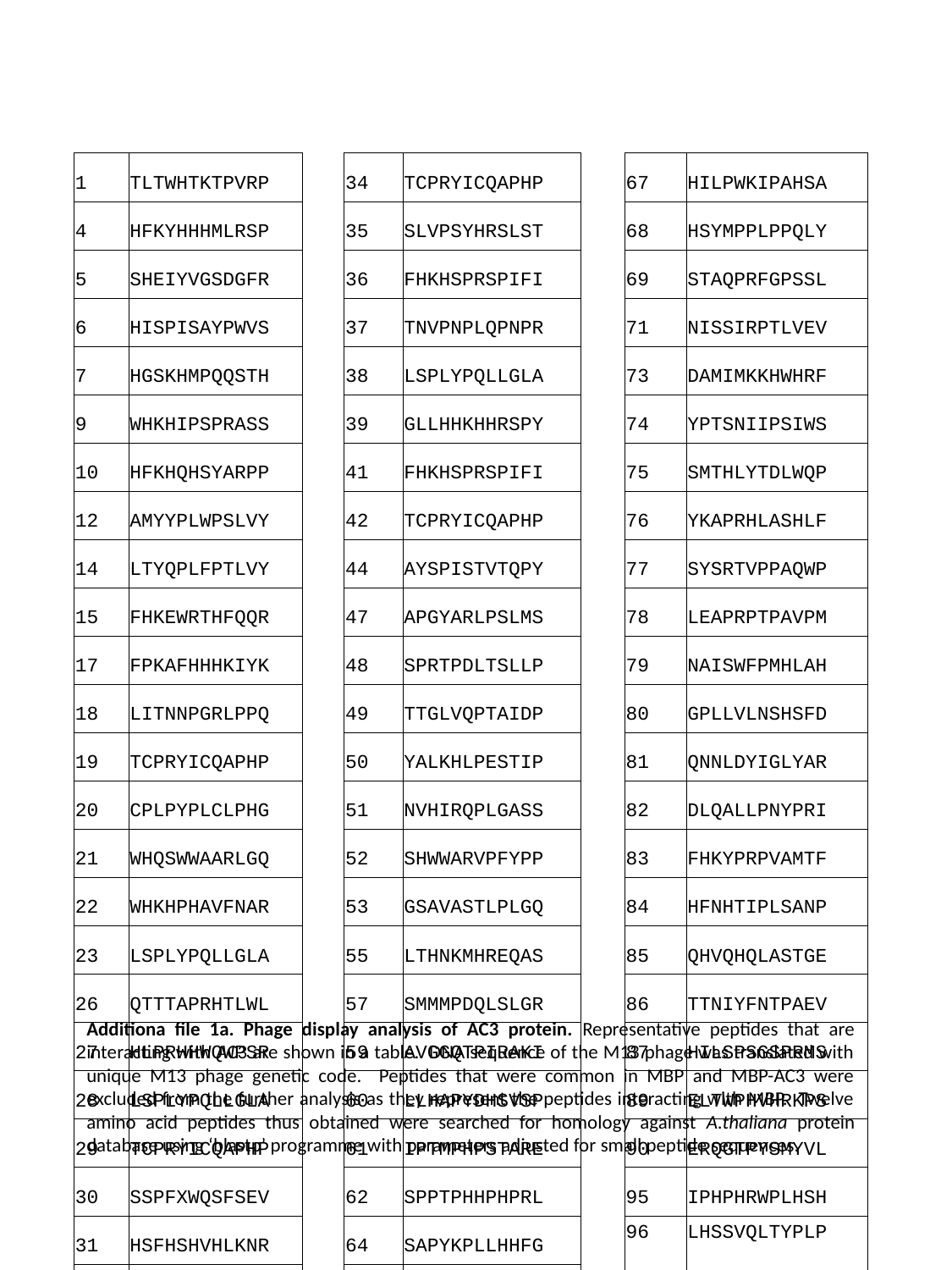

| 1 | TLTWHTKTPVRP | | 34 | TCPRYICQAPHP | | 67 | HILPWKIPAHSA |
| --- | --- | --- | --- | --- | --- | --- | --- |
| 4 | HFKYHHHMLRSP | | 35 | SLVPSYHRSLST | | 68 | HSYMPPLPPQLY |
| 5 | SHEIYVGSDGFR | | 36 | FHKHSPRSPIFI | | 69 | STAQPRFGPSSL |
| 6 | HISPISAYPWVS | | 37 | TNVPNPLQPNPR | | 71 | NISSIRPTLVEV |
| 7 | HGSKHMPQQSTH | | 38 | LSPLYPQLLGLA | | 73 | DAMIMKKHWHRF |
| 9 | WHKHIPSPRASS | | 39 | GLLHHKHHRSPY | | 74 | YPTSNIIPSIWS |
| 10 | HFKHQHSYARPP | | 41 | FHKHSPRSPIFI | | 75 | SMTHLYTDLWQP |
| 12 | AMYYPLWPSLVY | | 42 | TCPRYICQAPHP | | 76 | YKAPRHLASHLF |
| 14 | LTYQPLFPTLVY | | 44 | AYSPISTVTQPY | | 77 | SYSRTVPPAQWP |
| 15 | FHKEWRTHFQQR | | 47 | APGYARLPSLMS | | 78 | LEAPRPTPAVPM |
| 17 | FPKAFHHHKIYK | | 48 | SPRTPDLTSLLP | | 79 | NAISWFPMHLAH |
| 18 | LITNNPGRLPPQ | | 49 | TTGLVQPTAIDP | | 80 | GPLLVLNSHSFD |
| 19 | TCPRYICQAPHP | | 50 | YALKHLPESTIP | | 81 | QNNLDYIGLYAR |
| 20 | CPLPYPLCLPHG | | 51 | NVHIRQPLGASS | | 82 | DLQALLPNYPRI |
| 21 | WHQSWWAARLGQ | | 52 | SHWWARVPFYPP | | 83 | FHKYPRPVAMTF |
| 22 | WHKHPHAVFNAR | | 53 | GSAVASTLPLGQ | | 84 | HFNHTIPLSANP |
| 23 | LSPLYPQLLGLA | | 55 | LTHNKMHREQAS | | 85 | QHVQHQLASTGE |
| 26 | QTTTAPRHTLWL | | 57 | SMMMPDQLSLGR | | 86 | TTNIYFNTPAEV |
| 27 | HLPRHHWQWPSR | | 59 | AVGGQTPIRAKI | | 87 | HILSPSGSPRMS |
| 28 | LSPLYPQLLGLA | | 60 | LLHAPYDHSVSP | | 89 | ELTWPHVHRKPS |
| 29 | TCPRYICQAPHP | | 61 | IPTMPHPSTARE | | 90 | ERQGTPYSMYVL |
| 30 | SSPFXWQSFSEV | | 62 | SPPTPHHPHPRL | | 95 | IPHPHRWPLHSH |
| 31 | HSFHSHVHLKNR | | 64 | SAPYKPLLHHFG | | 96 | LHSSVQLTYPLP |
| 32 | HISPISAYPWVS | | 65 | IQSGTPHPPLRS | | | |
| 33 | WHSSWKSRVVPT | | 66 | QSMLGFISVSAK | | | |
Additiona file 1a. Phage display analysis of AC3 protein. Representative peptides that are interacting with AC3 are shown in a table. DNA sequence of the M13 phage was translated with unique M13 phage genetic code. Peptides that were common in MBP and MBP-AC3 were excluded from the further analysis as they represent the peptides interacting with MBP. Twelve amino acid peptides thus obtained were searched for homology against A.thaliana protein database using ‘blastp’ programme with parameters adjusted for small peptide sequences.

## Slide 2
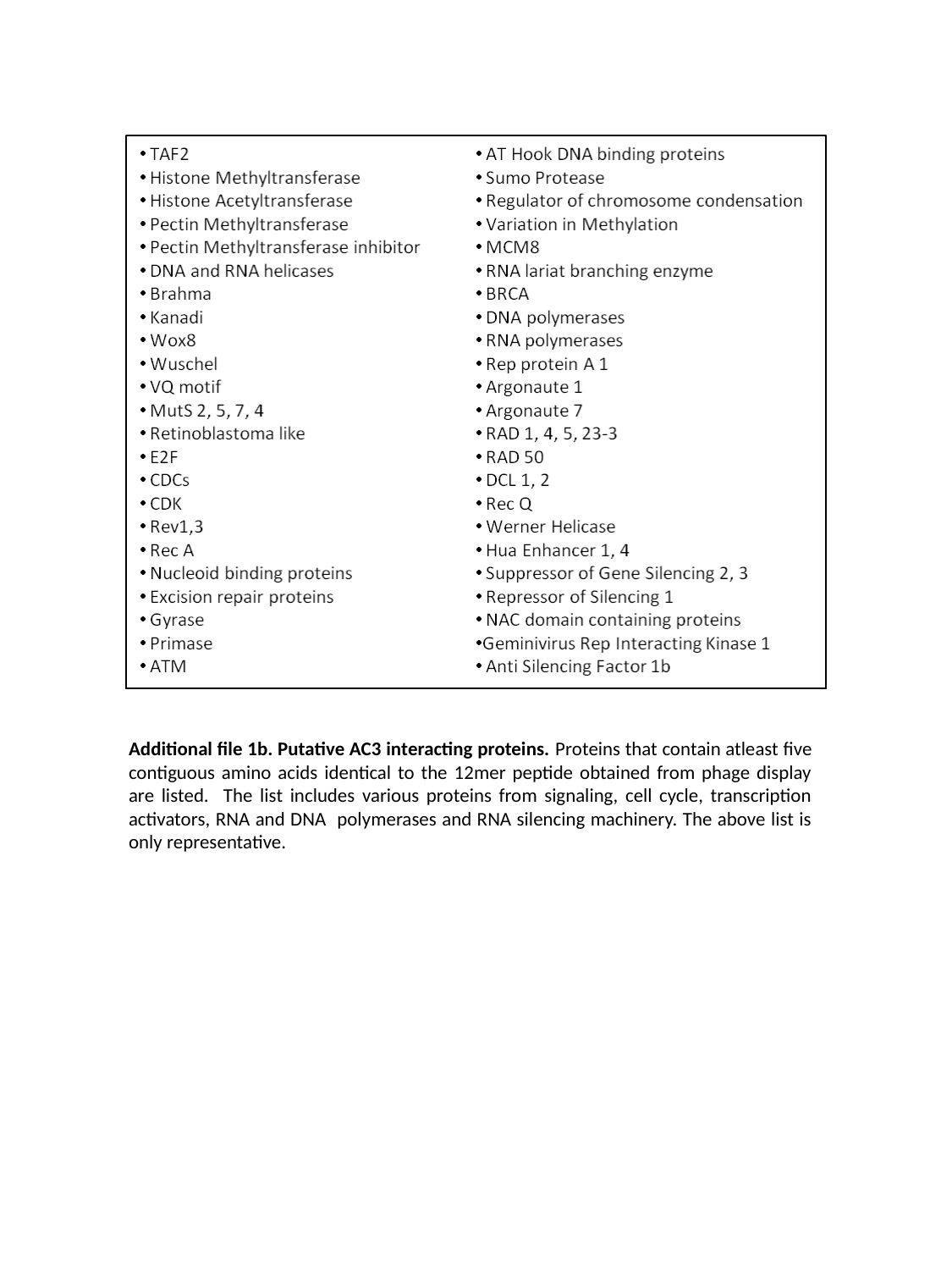

Additional file 1b. Putative AC3 interacting proteins. Proteins that contain atleast five contiguous amino acids identical to the 12mer peptide obtained from phage display are listed. The list includes various proteins from signaling, cell cycle, transcription activators, RNA and DNA polymerases and RNA silencing machinery. The above list is only representative.
