# Supplementary material for: The presence of tomato leaf curl Kerala virus AC3 protein enhances viral DNA replication and modulates virus induced gene-silencing mechanism in tomato plants
Source: Virol J. 2011 Apr 18;8:178. doi: 10.1186/1743-422X-8-178 (PMC3102638; doi:10.1186/1743-422X-8-178)
Supplement: Additional file 2 — Replication efficiency of ToLCKeV in yeast. Yeast cells were transformed with wild type YCp50 plasmid or YCp-CRAC3 (ToLCKeV) and incubated at 30°C for five days in Ura- medium. Yeast transformed with YCp50 grew normally while yeast transformed with YCp-CRAC3 (ToLCKeV) exhibited delayed growth. [file 1743-422X-8-178-S2.PPT]

## Slide 1
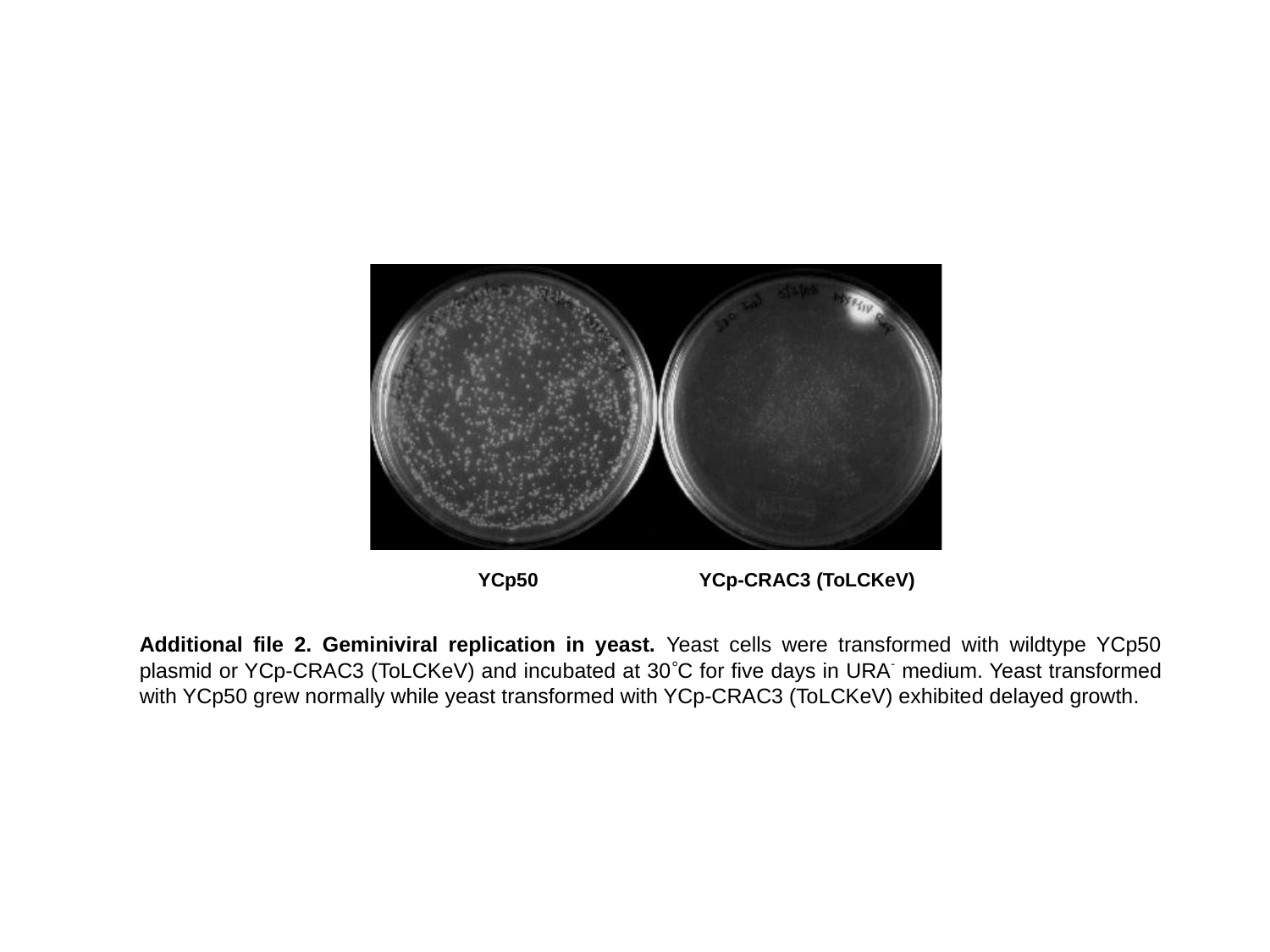

YCp50
YCp-CRAC3 (ToLCKeV)
Additional file 2. Geminiviral replication in yeast. Yeast cells were transformed with wildtype YCp50 plasmid or YCp-CRAC3 (ToLCKeV) and incubated at 30°C for five days in URA- medium. Yeast transformed with YCp50 grew normally while yeast transformed with YCp-CRAC3 (ToLCKeV) exhibited delayed growth.
